# ﻿Morphology and molecular phylogeny of *Mayamaea densestriata* sp. nov. (Bacillariophyceae), a new terrestrial species from Henan Province, China

**DOI:** 10.3897/phytokeys.268.171181

**Published:** 2025-12-15

**Authors:** Fei-Chao Du, Na Wei, Hao-Yu Li, Zhen-Quan Xu, Yu-Hang Li, Man Zhang

**Affiliations:** 1 College of Fisheries, Henan Normal University, Xinxiang 453007, China; 2 Engineering Technology Research Center of Henan Province for Aquatic Animal Cultivation, Henan Normal University, Xinxiang 453007, China; 3 Laboratory of Marine Organism Taxonomy and Phylogeny, Qingdao Key Laboratory of Marine Biodiversity and Conservation, Institute of Oceanology, Chinese Academy of Sciences, Qingdao, 266071, China

**Keywords:** China, Mayamaea, morphology, new species, phylogeny, terrestrial diatoms

## Abstract

A new terrestrial diatom species, *Mayamaea densestriata***sp. nov.**, is described from moist agricultural soil in Henan Province, China. Morphologically, it exhibits the typical diagnostic features of the genus *Mayamaea*, including small, elliptical to linear-elliptical valves, areolae externally occluded with hymenes, uniseriate radiate striae, and a distinct sternum. *M. densestriata* closely resembles six congeners, including *M. atomus*, *M. terrestris*, *M. fossalis*, *M. vietnamica*, *M. arida*, and *M. permitis*, but can be clearly distinguished by its lanceolate axial area fusing with a transversely expanded central area, higher stria density, and a notably greater number of areolae per stria. Phylogenetic analyses based on concatenated SSU rDNA and *rbc*L gene sequences strongly support the placement of *M. densestriata* within the genus *Mayamaea*. Furthermore, the overall topology also confirms the monophyly of *Mayamaea*, which is resolved as sister to a clade encompassing species of *Pinnularia* and *Caloneis*, thereby supporting its affiliation within the family Pinnulariaceae.

## ﻿Introduction

The genus *Mayamaea* Lange-Bertalot was established (Lange-Bertalot 1997) to accommodate 13 small-celled species formerly assigned to *Navicula* sensu lato, with *M. atomus* (Kützing) Lange-Bertalot designated as the type species. The genus *Mayamaea* represents an ecologically significant group of pennate diatoms that is abundant in freshwater and semi-terrestrial environments (Lange-Bertalot 1997; Morales and Manoylov 2009; Barragán et al. 2017; Lange-Bertalot et al. 2017; Kezlya et al. 2020). Members of *Mayamaea* are characterized by elliptical valve outlines, areolae externally occluded with hymenes, uniseriate striae, and a distinct central sternum (Kezlya et al. 2020). The structure and external positioning of hymenes serve as an important diagnostic feature, distinguishing *Mayamaea* from morphologically similar genera such as *Eolimna* Lange-Bertalot & W. Schiller and *Sellaphora* Mereschkowsky (Barragán et al. 2017). To date, approximately 34 taxa have been described within the genus *Mayamaea*, comprising 31 accepted species, two varieties, and one forma (Guiry and Guiry 2025). Although the genus exhibits a cosmopolitan distribution, most described species have been reported from temperate regions in Europe, with additional occurrences documented in Asia (Barragán et al. 2017; Bagmet et al. 2021; Vidaković et al. 2023). Notably, a recent study has shown that some *Mayamaea* species may prefer cold and oligotrophic habitats, suggesting broader ecological variability within the genus (Levkov et al. 2023). In China, nine species have been recorded thus far, including *M. disjuncta* (Hustedt) J.Y. Li & Y.Z. Qi, *M. excelsa* (Krasske) Lange-Bertalot, *M. agrestis* (Hustedt) Lange-Bertalot, *M. fossalis* (Krasske) Lange-Bertalot, *M. ingenua* (Hustedt) Lange-Bertalot & G. Hofmann, *M. muraliformis* Lange-Bertalot, *M. permitis* (Hustedt) K. Bruder & Medlin, *M. recondita* (Hustedt) Lange-Bertalot, and *M. fukiensis* (Skvortzov) J.Y. Lin & Y.Z. Qi (Guiry and Guiry 2025).

In recent decades, *Mayamaea* has attracted increasing attention due to its notable ecological plasticity, particularly its ability to thrive under fluctuating moisture conditions in environments such as humid soils, moss layers, and ephemeral aquatic habitats (Kulikovskiy 2006; Kulikovskiy et al. 2016; Barragán et al. 2017; Kezlya et al. 2020; Bagmet et al. 2021). Several species, including *M. lacunolaciniata* (Lange-Bertalot & Bonik) Lange-Bertalot, *M. muraliformis*, *M. terrestris* N. Abarca & R. Jahn, *M. petersenii* Barragán, Ector & C.E. Wetzel, *M. vietnamica* Glushchenko, Kezlya, Kulikovskiy & Kociolek, and *M. arida* (Bock) Lange-Bertalot, have been described from terrestrial or semi-terrestrial habitats (Ohtsuka 2001; Barragán et al. 2017; Foets et al. 2020; Kezlya et al. 2020; Bagmet et al. 2021; Vidaković et al. 2023). In addition, some traditionally aquatic species, such as *M. atomus*, have also been documented in soil environments. For instance, *M. atomus* has been reported from rice paddy soil in central Japan (Ohtsuka 2001) as well as dominating cultivated soils in Poland (Noga et al. 2014). In Luxembourg, *M. atomus* co-occurs with other soil-associated *Mayamaea* taxa, such as *M. agrestis*, *M. permitis*, and *M. fossalis*, across a variety of soil types, including agricultural fields and grasslands (Foets et al. 2020). Collectively, these findings suggest that terrestrial habitats may harbor a substantially greater diversity of *Mayamaea* than is currently recognized, but related investigations remain limited.

In this study, we describe a new species, *Mayamaea densestriata* sp. nov., isolated from moist agricultural soil in Henan Province, China. Morphological features are documented using both light microscopy (LM) and scanning electron microscopy (SEM). Phylogenetic analyses based on SSU rDNA and *rbc*L gene sequences further elucidate its taxonomic position. This discovery contributes to the growing catalog of terrestrial *Mayamaea* diversity and highlights the ecological breadth of the genus in soil environments.

## ﻿Materials and methods

### ﻿Sampling, cultivation, and morphological observation

A single moist soil sample was collected on 10 May 2024 from an active agricultural field located in Yuanyang County, Henan Province, China (35°05.40'N, 114°08.59'E). Single cells of diatoms were isolated using capillary pipettes under an inverted microscope and cultivated in CSI medium (composition following Nakayama et al. 2011). Non-axenic unialgal cultures were maintained at 20–22 °C under a light intensity of 80–100 μmol photon/m^2^/s, with a light/dark cycle of 12:12 h.

Prior to morphological examination, approximately 5 mL of culture was fixed with 2.5% glutaraldehyde and cleaned with hydrogen peroxide following the procedure of Trobajo and Mann (2019). For LM observation, cleaned frustules were mounted on glass slides using Mountmedia (Wako Pure Chemical Industries, Ltd., Osaka, Japan). Observations were performed using a Zeiss Imager Z2 microscope (Carl Zeiss Microimaging GmbH, Jena, Germany) equipped with differential interference contrast (DIC) optics. For SEM observations, cleaned material was placed on coverslips, air-dried, and coated with a thin layer of osmium. SEM observations were conducted using a JEOL JSM-7800F field emission scanning electron microscope (JEOL Ltd., Tokyo, Japan).

### ﻿DNA extraction and sequencing

Total DNA of monoclonal cultures was extracted and sequenced following the method described in our previous study (Du et al. 2023). Algal cell pellets were obtained by centrifuging 30 mL of diatom cultures at 5,000 g for 5 min. Total DNA was extracted using the Plant Genomic DNA Kit (Tiangen Biotech Co., Ltd., Beijing, China). Two genetic markers were amplified: the 18S small subunit ribosomal DNA (SSU rDNA) sequence and the partial chloroplast-encoded large subunit of the Rubisco (*rbc*L) gene. PCR reactions were performed in a total volume of 25 μL containing 1 μL of template DNA, 12.5 μL of 2× Plant Direct Master Mix polymerase (Vazyme Biotech Co., Ltd., Nanjing, China), 0.5 μL of each primer (10 mmol L^−1^), and 10.5 μL of sterile distilled water. The SSU rDNA was amplified using primers SSU1 and ITS1DR (Medlin et al. 1988; Edgar and Theriot 2004), while the *rbc*L gene was amplified using primers *rbc*L 66+ and *rbc*L 1444– (Alverson et al. 2007; Ruck and Theriot 2011). PCR cycling conditions for both markers followed those described by Alverson et al. (2007). All primers used in this study are listed in Table 1.

**Table 1.** Primers used to amplify SSU rDNA and *rbc*L fragments from *M. densestriata*.

Name	Marker	Sequence (5′ to 3′)	Reference
*SSU*1	SSU	AACCTGGTTGATCCTGCCAGT	(Medlin et al. 1988)
ITS1DR	SSU	CCTTGTTACGACTTCACCTTCC	(Edgar and Theriot 2004)
*rbc*L 66+	*rbc*L	TTAAGGAGAAATAAATGTCTCAATCTG	(Alverson et al. 2007)
*rbc*L 1444-	*rbc*L	GCGAAATCAGCTGTATCTGTW G	(Ruck and Theriot 2011)

Amplicons were visualized on 1.5% agarose gels stained with GoldView (DingGuo ChangSheng Biotech Co., Ltd., Beijing, China). PCR products were purified using the TIANgel Midi Purification Kit (Tiangen Biotech Co., Ltd., Beijing, China) according to the manufacturer’s protocol. Bidirectional sequencing of both SSU rDNA and *rbc*L was conducted using the same primers at Sangon Biotech Co., Ltd. (Shanghai, China).

### ﻿Molecular phylogenetic analyses

To examine the phylogenetic position of the new *Mayamaea* species, a two-gene dataset (SSU rDNA–*rbc*L) was used to construct the phylogenetic tree. The dataset comprised 80 recognized species and 9 unidentified strains, among which 19 strains belonged to the genus *Mayamaea* (taxa and accession numbers are given in Suppl. material 1). Taxon sampling comprised all available species from genera within Pinnulariaceae and closely related families, with three araphid diatoms serving as outgroup taxa.

Sequence alignment was performed using MAFFT v7.313 (Katoh and Standley 2013), with the Q-INS-i algorithm applied to SSU rDNA to account for RNA secondary structure. The resulting alignments were trimmed using trimAl v1.4 with the “automated1” parameter (Capella-Gutiérrez et al. 2009). The final concatenated alignment consisted of 2,704 positions, including 1,426 columns from SSU rDNA and 1,278 from *rbc*L. PartitionFinder 2 (Lanfear et al. 2017) was employed to identify the best-fit substitution models and partitioning schemes for maximum likelihood (ML) and Bayesian inference (BI) analyses based on the Bayesian Information Criterion (BIC). The *rbc*L gene was partitioned by codon position. ML analysis was conducted using IQ-TREE v1.6.8 (Nguyen et al. 2015) with 1,000 ultrafast bootstrap replicates under default settings. BI analysis was performed in MrBayes v3.2.7 (Huelsenbeck and Ronquist 2001), running 10^7^ generations with sampling every 1,000 generations. The initial 25% of the trees were discarded as burn-in. Convergence was assessed based on the average standard deviation of split frequencies (< 0.01), and effective sample size (ESS) values (> 3,000) were computed using the R package RWTY (Warren et al. 2017). The final consensus tree topology and posterior probabilities for all branches were calculated using a majority-rule consensus approach. Phylogenetic trees were visualized and edited with FigTree v1.4.4 and Adobe Illustrator.

## ﻿Results

### ﻿ Mayamaea densestriata sp. nov.

Taxon classificationPlantaeNaviculalesNaviculaceae

C6855D2C-9732-5CFB-9662-12880C942FD4

Fig. 1, Table 2

#### Description.

**Figure 1.**
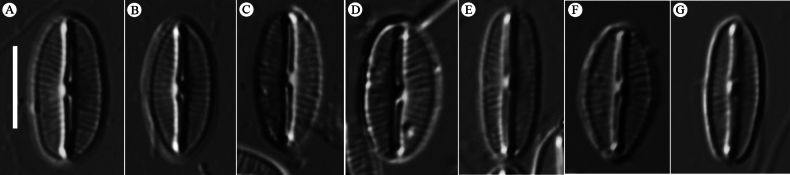
Valve face of *M. densestriata* under LM. **A.** Holotype; **B–G.** Valve size series. Scale bars: 5 μm (**A–G**).

In LM (Fig. 1A–G), valves small, elliptic to linear elliptic, with pronounced sternum and broadly rounded apices, 7.6–9.0 μm in length and 3.4–4.8 μm in width (n = 20). Raphe narrow, straight. Axial area lanceolate, broad towards valve center, fusing with transverse expanded central area. Central area wide, irregular, bordered on each margin by 7–10 shortened striae. The striae radiate throughout, 32–40 in 10 μm.

**Figure 2.**
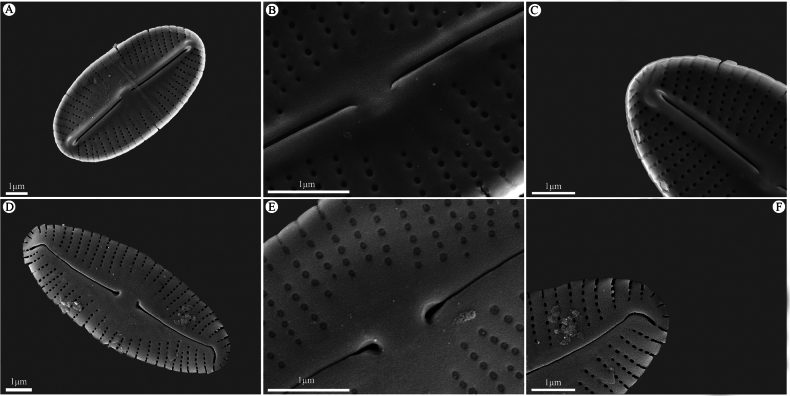
**A–F**SEM photographs of *M. densestriata*. **A.** Internal view of the whole valve; **B.** Internal valve central area showing wide, irregular, bordered margins with several shortened striae; **C.** Internal raphe fissures terminating in small helictoglossae at the apices; **D.** External view of the whole valve showing the terminal fissures slightly deflecting in one direction; **E.** External central area of the valve showing proximal raphe fissures ending in pore-like structures and slightly deflecting in one direction; **F.** Terminal fissures. Scale bars: 1 μm (**A–F**).

In SEM (Fig. 2), internally, raphe linear with thick distinct sternum (Fig. 2A). Central raphe endings slightly deflected to the same side of the valve (Fig. 2B). Distal raphe fissures terminated in small helictoglossae (Fig. 2C). Areolae small and round. Striae composed of 2–9 areolae. Central area wide. Externally, proximal raphe ends expanded and pore-like, surround by a shallow grove and slightly deflected in one direction (Fig. 2D, E). Terminal fissures bend markedly to the opposite direction of the proximal ends (Fig. 2F). Areolae small, round, and occluded by hymens. Striae composed of 6–8 round areolae and extended slightly onto the shallow margin of the valve.

#### Holotype.

The holotype is represented by the specimen illustrated in Fig. 1. The slide MBMCAS288335 is deposited in the Marine Biological Museum, Chinese Academy of Sciences (MBMCAS), Qingdao, China.

**Table 2.** Comparison of morphological features of *Mayamaea densestriata* with similar species. nd = not documented.

Characteristics	*M. densestriata*	*M. atomus*	*M. terrestris*	*M. vietnamica*	*M. arida*	*M. permitis*	*M. fossalis*
Valve outline	Elliptic to linear elliptic	Elliptic to linear elliptic	Narrow linear elliptical	Almost linear to elliptical and oval	Widely rounded	Elliptical	Elliptical
Length (μm)	7.6–9.0	8–13	7.0–8.7	9.1–10.5	4.8–9.0	6–9	9–12
Width (μm)	3.4–4.8	3.5–4.5	3.0–4.5	3.9–4.8	3.3–4.7	3–4	3–5
Axial area	Lanceolate, broad towards central area	Conforming with the sternum	Slightly broad, lanceolate from the middle of the valve	Tapers from the central area, becoming narrower towards the ends	Wide, well developed	Narrow, linear	Narrow-lanceolate, more rarely linear, differentiated from a central area
Central area	Transversely expanded, bordered on each margin by 7–10 shortened striae	Very small to absent, irregu- larly delimited by unequally shortened striae	Almost entirely lacking, central striae alternatingly shortened	More or less expressed, rounded to asymmetrical, rarely transversally elongated, and bordered on each margin by 3 shortened striae and / or 3 isolated areolae	Absent	Reduced and rounded	Distinct transversely-expanded central area due to some shortened striae
Striae	Uniseriate, radiate	Uniseriate, strongly radiate throughout	Uniseriate, radiate	Uniseriate, coarse, almost parallel at the valve centre, radial at the valve ends	Uniseriate, parallel in the central part, and radial in the ends	Uniseriate, radiate	Uniseriate, relatively coarse, radiate
Striae in 10 μm	32–40	18–22	22–26	19–22	24–32	30–36	16–21
Areolae per stria	2–9	1–8	1–6	1–3	1–2	nd	1–4
Habitat	Terrestrial	Freshwater, terrestrial	Terrestrial	Terrestrial	Freshwater, terrestrial	Freshwater, terrestrial	Freshwater, terrestrial
Reference	This study	(Mayama and Kobayasi 1988; Lange-Bertalot 2001; Lange-Bertalot et al. 2017)	(Zimmermann et al. 2014)	(Kezlya et al. 2020)	(Bagmet et al. 2021; Lange-Bertalot 1997)	(Bruder and Medlin 2008)	(Kulikovskiy 2006; Kulikovskiy et al. 2016; Lange-Bertalot 2001; Lange-Bertalot et al. 2017)

nd = not documented.

#### Type locality.

Surface of moist agricultural soil (35°05.40'N, 114°08.59'E) in Yuanyang County, Henan Province, China. The type material was collected by Feichao Du on 10 May 2024.

#### Etymology.

The specific epithet *Mayamaea densestriata* refers to the unusually high number of areolae per stria observed in this species. It highlights a key diagnostic feature that distinguishes it from its morphologically similar congeners.

#### Distribution and ecology.

*Mayamaea densestriata* is currently known only from its type locality. It is a terrestrial species associated with moist soil environments within cultivated agricultural landscapes.

#### Gene sequences.

The two sequences of *Mayamaea densestriata* have been deposited in the GenBank (SSU rDNA: PV733928, *rbc*L: PV740453).

#### PhycoBank registration.

http://phycobank.org/105610

##### ﻿Molecular phylogenetic analyses

**Figure 3.**
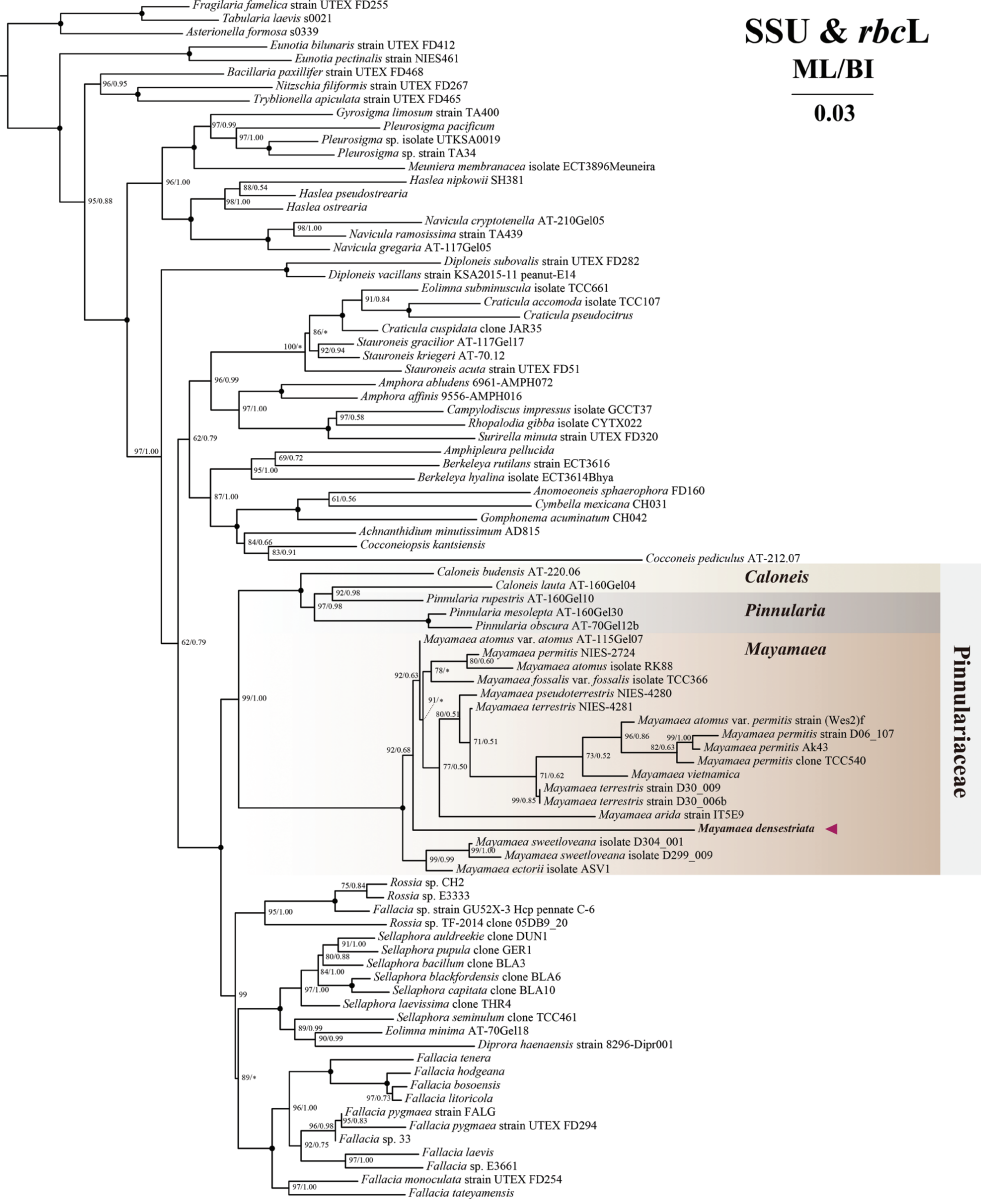
Phylogenetic trees using both maximum likelihood (ML) and Bayesian inference (BI) methods based on concatenated SSU rDNA and *rbc*L gene sequences. Nodal support values are presented as ML bootstrap percentages and Bayesian posterior probabilities, respectively. Only bootstrap values exceeding 50% are displayed. Solid circles denote full support in both analyses (ML = 100%, BI = 1.00), whereas asterisks mark topological discrepancies between the ML and BI trees.

The BLASTn search results showed that the SSU rDNA sequence of *Mayamaea densestriata* shares 89.37% identity with *M. permitis* (GenBank accession PV156110), while the *rbc*L gene exhibits 91.50% identity with M. atomus var. permitis (JN418670). Phylogenetic analyses based on concatenated SSU rDNA and *rbc*L genes place *M. densestriata* firmly within the well-supported clade comprising all currently recognized *Mayamaea* species (Fig. 3). Within this clade, *M. densestriata* occupies an independent branch and is placed at the basal position relative to a subclade including *M. terrestris*, *M. pseudoterrestris*, *M. arida*, *M. vietnamica*, M. atomus var. atomus, *M. permitis*, *M. atomus*, M. fossalis var. fossalis, and M. atomus var. permitis (ML = 92, BI = 0.68). Together, they are resolved as the sister to another *Mayamaea* subclade, including *M. sweetloveana* and *M. ectorii* with high support (ML = 100, BI = 1.00). The clade of *Mayamaea* is resolved as the sister group to a clade composed of *Pinnularia* Ehrenberg and *Caloneis* Cleve species (ML = 99, BI = 1.00), forming a monophyletic lineage of the family Pinnulariaceae.

## ﻿Discussion

### ﻿Morphological comparison with similar taxa

The newly described species *Mayamaea densestriata* sp. nov. has all the diagnostic features of the genus *Mayamaea*, including cells of small size, areolae externally occluded with hymenes, uniseriate striae, and a clearly well-developed sternum (Lange-Bertalot 1997). Morphologically, *M. densestriata* is closely comparable to several other taxa within the genus, such as *M. atomus*, *M. terrestris*, *M. fossalis*, *M. vietnamica*, *M. arida*, and *M. permitis* (Table 1). Among these, *M. densestriata* can be easily distinguished from *M. atomus*, *M. terrestris*, *M. vietnamica*, and *M. fossalis* by its significantly higher stria density (32–40 in 10 μm). *M. densestriata* differs from *M. arida* in terms of central area morphology and striae orientation. Specifically, *M. densestriata* possesses a wide, irregular, bordered central area and radiates striae across the valve face. In contrast, *M. arida* lacks the central area, and its striae are parallel in the central part of the valve. Additionally, *M. densestriata* displays a higher number of areolae per stria than *M. arida*. In terms of general outline and valve size, *M. densestriata* shows the greatest similarity to *M. permitis*, with both taxa exhibiting elliptical valves, comparable length and width ranges, and radiate striae with similarly high density. However, *M. densestriata* differs from *M. permitis* in having a lanceolate axial area that fuses with a transversely expanded central area, whereas *M. permitis* possesses a narrower axial area and a round to oval central area. These morphological differences support the recognition of *M. densestriata* as a distinct species.

### ﻿Phylogeny analysis

The molecular phylogenetic analysis conducted in this study provides strong support for the monophyly of the genus *Mayamaea*. While Kezlya et al. (2020) previously proposed a closer evolutionary relationship between *Mayamaea* and morphologically similar genera such as *Sellaphora*, *Eolimna*, and *Rossia* M. Voigt, our results resolve *Mayamaea* as the sister group to a clade containing species of *Caloneis* and *Pinnularia*. This finding supports the placement of *Mayamaea* within a monophyletic family-level lineage in Pinnulariaceae, consistent with the conclusions of Li et al. (2022).

Ecologically, species within *Mayamaea* can be broadly classified into three groups based on habitat preference: fully aquatic, semi-aquatic, and terrestrial environments (Bíró et al. 2022). Our phylogenetic reconstruction (Fig. 2) reveals that aquatic species form a well-supported monophyletic group, which is sister to a larger clade composed of semi-aquatic and terrestrial taxa. Notably, species inhabiting semi-aquatic and terrestrial environments do not form discrete clades but instead show a pattern of phylogenetic intermixing. This pattern, also observed in previous studies (Kezlya et al. 2020), suggests a continuum of ecological adaptation or a relatively recent divergence between these two habitat groups.

The absence of a clear phylogenetic boundary between semi-aquatic and terrestrial lineages may reflect ongoing ecological plasticity or incomplete lineage sorting associated with habitat transitions (Goetze et al. 2017). Such divergence is likely driven by selective pressures unique to edaphic environments, including desiccation, fluctuating moisture availability, light limitation, and nutrient heterogeneity. For example, terrestrial diatoms such as *Pinnularia borealis* Ehrenberg exhibit significantly greater tolerance to extreme temperatures and desiccation compared to aquatic counterparts (Souffreau et al. 2013; Hejduková et al. 2019), highlighting the importance of physiological adaptations in facilitating terrestrial colonization (Pinseel et al. 2020; Foets and Pinseel 2024).

In the future, a more comprehensive understanding of *Mayamaea* evolution will require integration of multiple approaches, including physiological experimentation, environmental sampling across hydrological gradients, and high-resolution genomic analyses. Such integrative strategies will be crucial for elucidating the molecular basis of habitat specialization and determining whether the current phylogenetic structure reflects evolutionary constraint, convergent adaptation, or responses to ecological opportunity.

## Supplementary Material

XML Treatment for Mayamaea densestriata

## ﻿Additional information

### Use of AI

No use of AI was reported.

### Author ORCIDs

Fei-Chao Du https://orcid.org/0009-0005-0674-5761

Yu-Hang Li https://orcid.org/0000-0002-5546-1774

### Data availability

All of the data that support the findings of this study are available in the main text or Supplementary Information.

## Supplementary materials

Supplementary material 1 GenBank accession of SSU rDNA and rbcL gene sequences derived from the species used in the phylogenetic analysis Data type pdf https://binary.pensoft.net/file/1490362This dataset is made available under the Open Database License (http://opendatacommons.org/licenses/odbl/1.0/). The Open Database License (ODbL) is a license agreement intended to allow users to freely share, modify, and use this Dataset while maintaining this same freedom for others, provided that the original source and author(s) are credited.Fei-Chao Du, Na Wei, Hao-Yu Li, Zhen-Quan Xu, Yu-Hang Li, Man Zhang
