# Supplementary material for: ﻿Morphology and molecular phylogeny of Mayamaea densestriata sp. nov. (Bacillariophyceae), a new terrestrial species from Henan Province, China
Source: PhytoKeys. 2025 Dec 15;268:33–44. doi: 10.3897/phytokeys.268.171181 (PMC12723397; doi:10.3897/phytokeys.268.171181)
Supplement: Supplementary material 1 — GenBank accession of SSU rDNA and rbcL gene sequences derived from the species used in the phylogenetic analysis [file phytokeys-268-033_article-171181__-s001.pdf]

Table S1. GenBank accession of SSU rDNA and *rbc*L gene sequences derived from the species used in the phylogenetic analysis.

Species	SSU	*rbc*L
Achnanthidium minutissimum AD815	KJ658398	KJ658382
Amphipleura pellucida	KC309477	KC309549
Amphora abludens 6961-AMPH072	KJ463426	KJ463456
Amphora affinis 9556-AMPH016	KJ463424	KJ463454
Anomoeoneis sphaerophora FD160	KJ011612	KJ011795
Asterionella formosa s0339	AB430595	AB430671
Bacillaria paxillifer strain UTEX FD468	HQ912627	HQ912491
Berkeleya hyalina isolate ECT3614Bhya	KJ577847	KJ577882
Berkeleya rutilans strain ECT3616	HQ912637	HQ912501
Caloneis budensis AT-220.06	AM502003	AM710470
Caloneis lauta AT-160Gel04	AM502039	AM710506
Campylodiscus impressus isolate GCCT37	KX120712	KX120599
Cocconeiopsis kantsiensis	OR712156	OR700023
Cocconeis pediculus AT-212.07	AM502010	AM710477
Craticula accomoda isolate TCC107	KF959652	KF959638
Craticula cuspidata clone JAR35	KM999000	KM999070
Craticula pseudocitrus	LC335883	LC335884
Cymbella mexicana CH031	KJ011624	KJ011807
Diploneis subovalis strain UTEX FD282	HQ912597	HQ912461
Diploneis vacillans strain KSA2015-11 peanut-E14	MH063462	MH064084
Diprora haenaensis strain 8296-Dipr001	KC954571	KC954572
Eolimna minima AT-70Gel18	AM501962	KM084930
Eolimna subminuscula isolate TCC661	KT072989	KT072935
Eunotia bilunaris strain UTEX FD412	HQ912599	HQ912463
Eunotia pectinalis strain NIES461	HQ912636	HQ912500
Fallacia bosoensis	MW917200	MW924346
Fallacia hodgeana	MW917203	MW924350
Fallacia laevis		MW924348
Fallacia litoricola	MW917201	MW924347
Fallacia monoculata strain UTEX FD254	HQ912596	HQ912460
Fallacia pygmaea strain FALG	KX257362	
Fallacia pygmaea strain UTEX FD294	HQ912605	HQ912469
Fallacia sp. 33	KJ961671	
Fallacia sp. E3661		EF143274
Fallacia sp. strain GU52X-3 Hcp pennate C-6	MH063467	MH064092
Fallacia tateyamensis	MW917199	MW924345
Fallacia tenera	MW917202	MW924349
Fragilaria famelica strain UTEX FD255	HQ912588	HQ912452
Gomphonema acuminatum CH042	KJ011671	KJ011853
Gyrosigma limosum strain TA400	KY320348	KY320287

Haslea nipkowii SH381	KY320351	KY320290
Haslea ostrearia	AY485523	HE663064
Haslea pseudostrearia	KY320350	KY320289
Mayamaea arida strain IT5E9		MZ400876
Mayamaea atomus isolate RK88	KF417670	
Mayamaea atomus var. *atomus* AT-115Gel07	AM501968	
Mayamaea atomus var. *permitis* strain (Wes2)f	JN418600	JN418670
Mayamaea densestriata	PV733928	PV740453
Mayamaea ectorii isolate ASV1		OP354489
Mayamaea fossalis var. *fossalis* isolate TCC366	KF959655	KF959641
Mayamaea permitis Ak43	LC648455	LC648450
Mayamaea permitis clone TCC540	KC736630	KC736600
Mayamaea permitis NIES-2724	LC648456	LC648446
Mayamaea permitis strain D06_107	FR873249	KM084957
Mayamaea pseudoterrestris NIES-4280	LC648451	LC648448
Mayamaea sweetloveana isolate D299_009	OR352038	OR355401
Mayamaea sweetloveana isolate D304_001	OR352039	OR355402
Mayamaea sweetloveana isolate D304_002		OR355403
Mayamaea terrestris NIES-4281	LC648452	LC648449
Mayamaea terrestris strain D30_006b	KM084908	KM084974
Mayamaea terrestris strain D30_009	KM084909	KM084975
Mayamaea vietnamica		MT024765
Meuniera membranacea isolate ECT3896Meuneira	KC309482	KC309554
Navicula cryptotenella AT-210Gel05	AM502015	AM710482
Navicula gregaria AT-117Gel05	AM501974	AM710440
Navicula ramosissima strain TA439	KY320363	KY320302
Nitzschia filiformis strain UTEX FD267	HQ912589	HQ912453
Pinnularia mesolepta AT-160Gel30	AM501994	AM710461
Pinnularia obscura AT-70Gel12b	AM501986	AM710452
Pinnularia rupestris AT-160Gel10	AM501992	AM710458
Pleurosigma pacificum	OQ437519	OQ473490
Pleurosigma sp. isolate UTKSA0019	KX981840	KX981822
Pleurosigma sp. strain TA34	KY320349	KY320288
Rhopalodia gibba isolate CYTX022	KX120675	KX120559
Rossia sp. CH2	AJ535144	
Rossia sp. E3333	EF151968	EF143281
Sellaphora auldreekie clone DUN1	EF151965	EF143276
Sellaphora bacillum clone BLA3	EF151980	EF143311
Sellaphora blackfordensis clone BLA6	EF151969	EF143282
Sellaphora capitata clone BLA10	EF151971	EF143295
Sellaphora laevissima clone THR4	EF151981	EF143309
Sellaphora pupula clone GER1	EF151984	EF143318
Sellaphora seminulum clone TCC461	KC736642	KC736613

Stauroneis acuta strain UTEX FD51	HQ912579	HQ912443
Stauroneis gracilior AT-117Gel17	AM501988	AM710454
Stauroneis kriegeri AT-70.12	AM501990	AM710456
Surirella minuta strain UTEX FD320	HQ912658	HQ912522
Tabularia laevis s0021	AB430610	AB430690
Tryblionella apiculata strain UTEX FD465	HQ912600	HQ912464
